# Caspase-mediated cleavage of actin and tubulin is a common feature and sensitive marker of axonal degeneration in neural development and injury

**DOI:** 10.1186/2051-5960-2-16

**Published:** 2014-02-07

**Authors:** Jennifer D Sokolowski, Kanchana K Gamage, Daniel S Heffron, Andrea C LeBlanc, Christopher D Deppmann, James W Mandell

**Affiliations:** 1 Department of Pathology, University of Virginia School of Medicine, Charlottesville, VA 22908, USA; 2 Medical Scientist Training Program, University of Virginia School of Medicine, Charlottesville, VA 22908, USA; 3 Neuroscience Graduate Program, University of Virginia School of Medicine, Charlottesville, VA 22908, USA; 4 Department of Biology, University of Virginia School of Medicine, Charlottesville, VA 22908, USA; 5 Department of Neurology and Neurosurgery, McGill University, University St, Montreal, QC, Canada

**Keywords:** Apoptosis, Pruning, Axon, Degeneration, Caspase, Cytoskeleton

## Abstract

**Background:**

Axon degeneration is a characteristic feature of multiple neuropathologic states and is also a mechanism of physiological neurodevelopmental pruning. The vast majority of *in vivo* studies looking at axon degeneration have relied on the use of classical silver degeneration stains, which have many limitations including lack of molecular specificity and incompatibility with immunolabeling methods. Because Wallerian degeneration is well known to involve cytoskeletal disassembly and because caspases are recently implicated in aspects of this process, we asked whether antibodies directed at caspase-generated neoepitopes of beta-actin and alpha-tubulin would be useful immunohistochemical markers of pathological and developmental axon degeneration.

**Results:**

Here we demonstrate that several forms of axon degeneration involve caspase-mediated cleavage of these cytoskeletal elements and are well-visualized using this approach. We demonstrate the generation of caspase-induced neoepitopes in a) an *in vitro* neuronal culture model using nerve growth factor-deprivation-induced degeneration and b) an *in vivo* model using ethanol-induced neuronal apoptosis, and c) during normal developmental pruning and physiological turnover of neurons.

**Conclusions:**

Our findings support recent experimental data that suggests caspase-3 and caspase-6 have specific non-redundant roles in developmental pruning. Finally, these findings may have clinical utility, as these markers highlight degenerating neurites in human hypoxic-ischemic injury. Our work not only confirms a common downstream mechanism involved in axon degeneration, but also illuminates the potential utility of caspase-cleavage-neoepitope antibodies as markers of neurodegeneration.

## Background

Axon degeneration occurs not only after injury but also during normal central nervous system (CNS) development. Degeneration may be initiated due to lack of trophic signals, presence of toxins, or trauma [1,2]. The upstream factors involved in axon degeneration are different depending on the context or the mode of injury; but regardless of the instigating stimulus, the process is an active, orchestrated event as opposed to a passive phenomenon [3].

There are many similarities between programmed cell death, or apoptosis, and the process of axon degeneration. Apoptotic signals may arise from a variety of stimuli, but both the extrinsic and intrinsic pathways converge to activate caspases, a group of proteolytic enzymes that cleave their substrates at specific residues [4]. Activated caspases are responsible for cleaving many proteins, including structural and repair proteins, and different executioner caspases, for example caspase-3 and caspase-6, have distinct, non-redundant roles [5]. Degradation of proteins, including cytoskeletal elements, leads to morphological changes such as blebbing of the membrane, and the release of apoptotic bodies, or membrane-bound vesicles [6,7]. Elucidating the role of caspases in axon degeneration has been of interest to the field, and studies have attempted to determine whether axon degeneration during apoptosis utilizes the same mechanisms as those that occur during selective axon pruning [8-10]. Axon degeneration can be induced *in vitro* in cultured sympathetic neurons via nerve growth factor (NGF) deprivation. NGF deprivation has been widely used as an *in vitro* model to induce axon degeneration and is thought to model many aspects of developmental pruning [1]. Groups have used this model to show that degeneration of axons during apoptosis occurs via different mechanisms than during selective pruning of parts of axons [8,9]. In order to induce *selective* degeneration of axons, neurons are grown in devices such as microfluidic chambers that allow compartmentalization of distal axons and dendrite/cell bodies, which enables selective NGF-deprivation of axons exclusively. Early work suggested that caspases had no role in selective axon degeneration, as caspase inhibition was not able to prevent degeneration after local deprivation of NGF [9]. However, work since then has implicated a role for caspase-6 in selective degeneration. Caspase-6 is activated in degenerating neurons and caspase-6 deficiency protected axons from selective degeneration after local NGF deprivation [8,11], although it did not prevent degeneration after whole-cell deprivation [8].

Recent work using genetic deletion of caspase-3 and caspase-6 indicated that both are required for NGF-deprivation induced axon degeneration as well as for proper developmental pruning of retinocollicular projections [10]. Endogenous caspase inhibitors also play a role, as XIAP-deficient mice have stunted dermal innervation [12]. Thus, caspases play a critical role in axonal pruning, in addition to their well-known role in apoptosis.

Ethanol exposure during embryonic and early postnatal ages induces substantial neuronal apoptosis [13]. Because substantial brain development (and a period of susceptibility to ethanol injury) occurs postnatally in mice, ethanol exposure at postnatal day 7 is a widely utilized model of fetal ethanol exposure in humans and has served as a powerful model to understand molecular mechanisms of neuronal apoptosis [13-17]. Ethanol-induced apoptosis is known to involve the mitochondrial pathway, as BAX and PUMA deficient animals are protected from caspase activation after acute ethanol injury [14]. Therefore, we used this model to study neuronal and axonal degeneration *in vivo*.

During degeneration, the axon blebs and fragments. This blebbing and fragmentation is dependent on cytoskeletal disassembly and has been hypothesized to be controlled by factors that regulate microtubule stability, such as microtubule-associated proteins [3]. The downstream mechanisms that cause cytoskeletal degradation in axons have not been fully elucidated, but ultimately, cytoskeletal degradation appears to be a point of convergence for axon degeneration induced by a variety of mechanisms.

We investigate this common convergence point using experimental models and human neuropathological specimens. We demonstrate that antibodies against caspase-generated neoepitopes of beta-actin and alpha-tubulin are sensitive and specific indicators of caspase-mediated cytoskeletal degradation. These markers indicate that axonal degeneration involves caspase-mediated cleavage of actin and tubulin. These cleavage events occur in a variety of contexts involving axon degeneration, including a) *in vitro* in neuronal cultures induced to undergo apoptosis via nerve growth factor deprivation, b) *in vivo* after ethanol-induced apoptosis as well as during developmental apoptosis and physiological turnover of neurons, and c) in human brain after acute or subacute hypoxic-ischemic injury.

## Methods

### Tissue processing

All animal procedures were according to NIH guidelines and approved by the University of Virginia Animal Care and Use Committee. Fifteen male or female C57/BL6 mice (Charles River) at either postnatal day 7 (3-5 g) or at 8–12 weeks (18-28 g) of age were used for this study. Fifteen rats (Sprague–Dawley, Harlan) at postnatal day 0–3 were used for neuron culture. Animals were housed in standard polypropylene cages with corncob bedding with food and water *ad libitum*. Adults were group housed (2–5 animals per cage) and pups were maintained with the dam. The colony was maintained on a 12:12 light: dark cycle in a temperature-controlled room. For tissue harvest, animals were anesthetized with a lethal dose of pentobarbital. Whole embryos were harvested from one pregnant female mouse at E14.5, and were post-fixed overnight in 4% paraformaldehyde. Ethanol experiments were done in postnatal mice and olfactory bulb tissue was collected from adult mouse brain. Brains from postnatal and adult mice were harvested and fixed in either 4% paraformaldehyde or 70% ethanol. Tissue was processed into paraffin by standard methods. Tissue from BAX knockout and PUMA knockout mice was provided by Dr. Kevin Roth (University of Alabama-Birmingham). For analysis of human specimens, archival paraffin-embedded brain tissue was obtained from autopsies from 5 patients who had been diagnosed with acute or subacute brain infarct.

### Caspase enzymatic assay and western blot

To prepare synaptosomes, cortex from three P7 mice was homogenized in Locke’s buffer with ten strokes of a tight-fitting glass Dounce tissue grinder. The homogenate was centrifuged at 500 *g* for 10 min and the resulting supernatant was centrifuged at 12,000 *g* for 15 minutes. The pellet was resuspended in Locke’s buffer and centrifuged at 12,000 *g* for 15 minutes to obtain the final pellet containing the synaptosome-enriched fraction. The caspase enzymatic assay was performed as previously described [18]. Laemmli sample buffer was added to synaptosomes and 50 ug of protein was loaded into each lane and separated by electrophoresis using standard procedures. Gels were transferred to a PVDF membrane for 90 min with a semidry transfer apparatus and treated with blocking reagent (LI-COR block; LI-COR, Lincoln NE) for 1 hour and then probed with primary antibodies overnight. Antibodies used were the following: TubulinΔCsp6 (LeBlanc lab, 1:20,000), alpha-tubulin (clone DM1A, 1:10,000), fractin (Millipore, 1:1000). For visualization, blots were incubated with fluorescent secondary antibodies (LI-COR, 1:2000) for 1–2 hours and imaged on a LI-COR Odyssey infrared scanner.

### Cell culture

Neurons for NGF deprivation were obtained by acute dissection and enzymatic dissociation of superior cervical ganglia from fifteen postnatal (P0-P3) rats. Neurons were plated in Poly-D-Lysine/Laminin-coated coverslips with compartmentalized microfluidic devices with DMEM supplemented with 10% FBS, penicillin/streptomycin (1 U/ml), and 40 ng/ml NGF purified from mouse salivary glands [19]. After 24–48 hours, 5 μM Ara-C was added to the culture media for 48 hours to reduce glial contamination. For microfluidic devices, neurons were given time to project their axons to the outer chamber (5–7 days *in vitro*).

### NGF-deprivation

For these experiments, there were three groups: NGF-replete neurons, NGF-deprived neurons, and NGF-deprived neurons treated with caspase inhibitor. There were 4–6 animals used per group. Neurons were either maintained in NGF or for NGF deprivation, cultures were rinsed three times with medium lacking NGF and then maintained in NGF-deficient media containing a neutralizing antibody to NGF (Millipore). For pan-caspase inhibition, neurons were treated with 100 μM of ZVAD (Enzo Life Sciences) concurrent with NGF deprivation. Neurons were fixed by incubating in 4% paraformaldehyde for 15 minutes, washing with PBS twice. Cells were then stained.

### Ethanol treatment in vivo

The groups used for this experiment were wild-type saline-treated controls, wild-type ethanol-treated mice, and BAX-knockout ethanol-treated mice. There were 2–3 animals for each group. Ethanol (20% solution in 0.9% saline) or .9% saline was injected subcutaneously into postnatal day 7 (P7) pups as a at 15.9 μL/g body weight. It was administered twice, 2 hours apart as previously described [14]. Brain tissue was harvested 6 or 24 hours after the first injection.

### Immunolabeling

For immunocytochemistry, cells were fixed for 15 minutes in 4% paraformaldehyde, rinsed in PBS then incubated for one hour in blocking solution (2% horse serum, 0.1% Tween in PBS) prior to overnight incubation with primary antibody (4°C, diluted in block). For tissue staining, paraffin-embedded sections were dewaxed, rehydrated, subjected to antigen retrieval (Tris-EDTA pH 9, 12 min over a boiling water bath), quenched (15 min, .6% H_2_O_2_ in dH_2_O) and blocked (1 hour) prior to incubation with primary (diluted in block, incubated overnight at 4°C).

### Antibodies

The antibodies used were: TubulinΔCsp6 (LeBlanc lab, 1:2000), fractin (Millipore, 1:1000), CC3 (Cell Signaling, 1:100), NFM (DSHB, clone 2H3, 1:2000), β3-tubulin (Tuj1, 1:500), iba1 (Wako, 1:500). Immunoperoxidase detection was performed using the ImmPress polymeric peroxidase reagents (Vector). Diaminobenzidine (Dako) 1 mg/ml plus 0.02% hydrogen peroxide was applied for 3–5 min. Immunofluorescence detection was performed using secondary antibodies conjugated to Alexa-488 and Alexa-546 dyes (Invitrogen, 1:2000) and DAPI (1 μg/mL) (diluted in block, incubated for 1 hour at room temperature). Tyramide Signal Amplification was performed after labeling with primary antibody. Sections were incubated with a biotinylated secondary antibody diluted in block (1:400, Vector), then an ABC kit (Vector) was used according to vendor instructions. Sections were then stained with Cy3-conjugated tyramine according to TSA kit instructions (NEN Life Sciences).

### Image analysis

Immunofluorescence images were acquired with an Olympus BX40 upright microscope and a Scion Firewire CCD camera (Scion, Frederick, MD). Exposure settings were adjusted for each stain to ensure that the intensity of all the pixels within the images were within the dynamic range for acquisition, and the same settings were used for each treatment condition. Fields with similar axon densities were chosen. An image in each channel was acquired for each field.

Cell profiler (Broad Institute, [20]) was used for automated analysis of images. Pipelines were set up to identify Tuj1-positive axons as follows: automated threshold settings in the ‘IdentifyPrimaryObjects’ module were adjusted so that Tuj1-positive areas were identified as objects; the fidelity of the masks for each image was confirmed by a blinded observer and the same threshold technique was used for all treatment conditions. The mean fluorescence intensity of fractin and TubulinΔCsp6 images were measured within the objects (axons) by using the ‘MeasureImageIntensity’ module, selecting the option to ‘measure the intensity only from areas enclosed by objects.’ Data was graphed and analyzed using Graphpad Prism (La Jolla, CA).

## Results

### Recombinant caspases are sufficient to generate fractin and cleaved tubulin: demonstration of neoepitope antibody specificity

**Figure 1:**
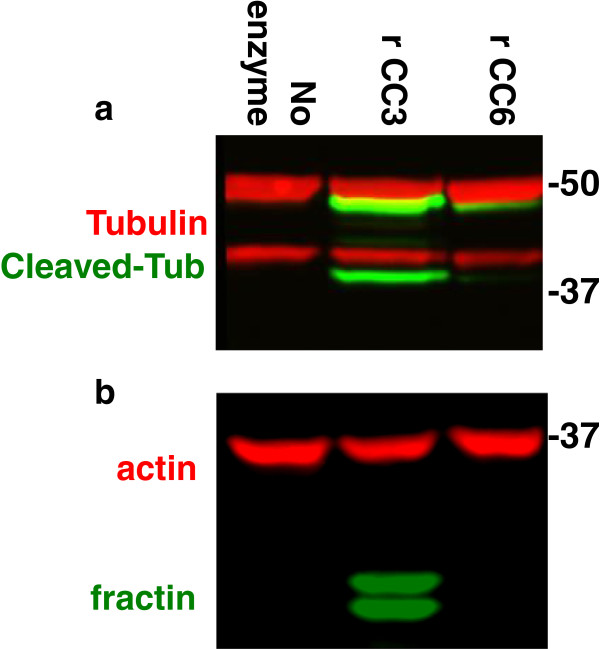
**Demonstration of specificity of caspase cleavage neoepitope antibodies to actin and tubulin.***In vitro* cleavage of the substrates was performed via incubation of either recombinant active caspase-3 or −6 with permeabilized brain-derived synaptosomes. A western blot was performed using lysate from this preparation. Blot **(a)** was colabeled with general anti-alpha-tubulin (tubulin, red) and TubulinΔCsp6 (Cleaved-Tub, green). Blot **(b)** was colabeled with anti-actin (red) and anti-fractin (green). Recombinant cleaved-caspase-3 (rCC3) generates both cleaved tubulin **(a)** and fractin **(b)**, whereas recombinant cleaved-caspase-6 (rCC6) only generates only cleaved tubulin **(a)**.

The fractin and TubulinΔCsp6 antibodies are specific to the caspase-cleaved fragments of beta-actin (cleaved by caspase-3 at D244 [21] and alpha-tubulin (cleaved by caspase 6 at D438 [22], respectively. We performed an *in vitro* enzymatic assay in order to test whether caspases are sufficient to generate these neoepitopes. We harvested synaptosomes from mouse brain and incubated them with recombinant caspases. We found that both caspase-3 and caspase-6 are capable of generating the epitope recognized by TubulinΔCsp6, whereas fractin is only produced by caspase-3 (Figure 1). For alpha-tubulin, we observed a doublet on western blot (Figure 1a), and we suspect that the lower band in the doublet may represent the α3 subunit of tubulin, which is known to be enriched in synaptosomes and has faster electrophoretic mobility [23]. The doublet seen with fractin (Figure 1b) may represent multiple cleavage products created by caspase-3. The no enzyme control shows the epitopes are not present in the control synaptosome preparation, indicating that they are specifically generated in the presence of caspases.

### NGF-withdrawal-induced apoptosis leads to cleavage of actin and tubulin in axons

**Figure 2:**
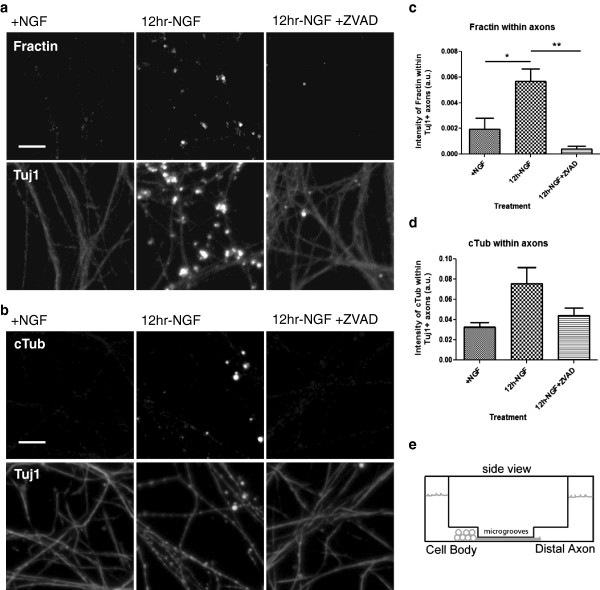
**NGF-deprivation induces caspase-mediated cleavage of actin and tubulin. (a, b)** NGF-replete (+NGF) cultured sympathetic neurons are viable and axons are intact, as visible with β_3_ tubulin (Tuj1) staining. NGF-withdrawal-induced apoptosis causes axon degeneration by 12 hours that is marked with antibodies against cleaved actin (fractin) **(a)** or TubulinΔCsp6 (cTub) **(b)** and is suppressed by caspase inhibition using ZVAD. Intensity of fractin **(c)** and TubulinΔCsp6 **(d)** staining in axons was measured in arbitrary fluorescence units (a.u.). A diagram of the microfluidic chamber used is shown in **(e)**. Data represents three independent experiments. One-way ANOVA, *p < .05, **p < .01. Scale bar = 10 μm.

We next employed an NGF-deprivation model to induce axon degeneration in cultured sympathetic neurons. The neurons were grown in microfluidic chambers in order to separately visualize the axons and the cell bodies (Schematic diagram, Figure 2e). NGF-replete neurons had little fractin or TubulinΔCsp6 labeling in the axonal compartment. However, 12 hours of global NGF-deprivation leads to fractin (Figure 2a) and cleaved tubulin (Figure 2b) production in axons. As expected, this staining corresponded with an increase in blebbing and fragmentation of the axons, confirmed by β_3_-tubulin (Tuj1) staining.

Caspase activity is sufficient to cleave actin and tubulin as demonstrated in our *in vitro* cleavage experiments (Figure 1). To test the necessity of caspase activity for generation of cleaved neoepitopes, we performed NGF-deprivation in the presence of a pan-caspase inhibitor (ZVAD). Of note, there is data to suggest that ZVAD does not effectively inhibit caspase-6 [24]. We quantified the fluorescence intensity of staining within axons. We found that 12 hours of NGF-deprivation leads to significant fractin production and incubation with ZVAD prevents the increase in staining (Figure 2c). TubulinΔCsp6 staining trended in the same direction (Figure 2d). Overall, this indicates that caspases are involved in axon degeneration downstream of trophic-factor-withdrawal.

### Cleavage of actin and tubulin occurs after induction of apoptosis

**Figure 3:**
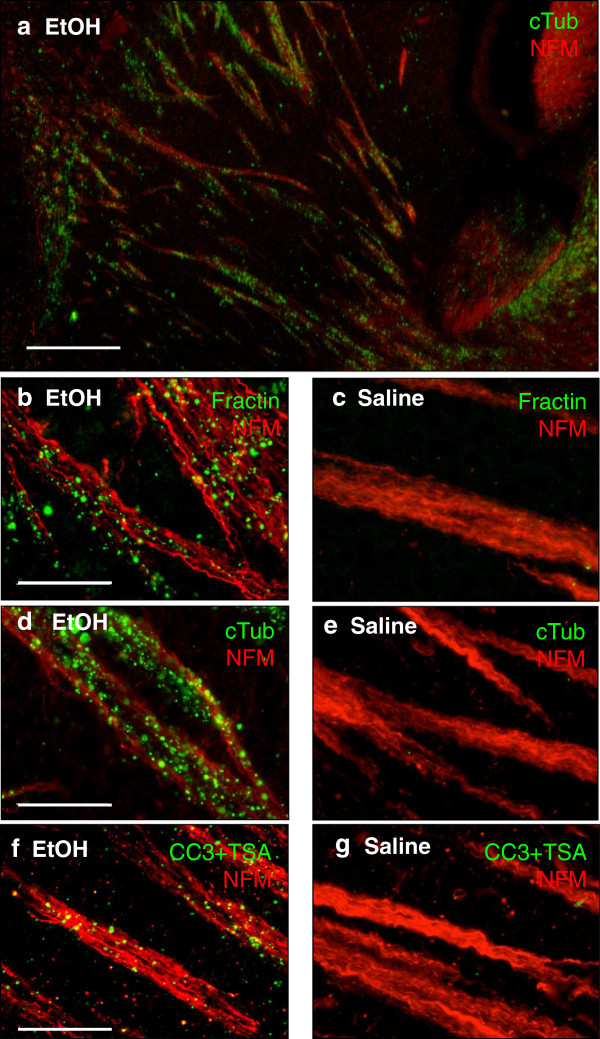
**Fractin and TubulinΔCsp6 labeling highlight degenerating axon tracts after ethanol-induced apoptosis. (a)** Low power image of co-labeling in the striatum using anti-tubulinΔCsp6 (cTub; green) along with anti-neurofilament (NFM; red) reveals that tubulinΔCsp6 localizes to axon tracts 24 hours post-ethanol treatment. **(b-c)** High power image of co-labeling in the striatum using fractin or TubulinΔCsp6 (green) along with a marker for neurofilaments (red) reveals that both fractin and TubulinΔCsp6 highlight blebbing material within axon tracts after ethanol-induced apoptosis **(b,d)** and are absent in control (saline-treated) brain **(c,e)**. **(f-g)** While ethanol-induced apoptosis led to CC3-positive cell bodies, CC3 was not usually seen in axon fibers via conventional staining techniques. Tyramide signal amplification (TSA) was required for visualization of CC3 in axon fibers after ethanol-induced apoptosis (shown are striatal pencil fibers). CC3 with TSA (CC3 + TSA) was only detected in tissue after ethanol-induced apoptosis **(f)**, and CC3 staining was not seen in control tissue **(g)**. Scale in **(a)** is 200 μm. Scale in **(b-g)** is 50 μm.

We used an animal model of fetal ethanol exposure in order to visualize axonal degeneration after induction of neuronal apoptosis *in vivo*[13]*.* We injected postnatal day 7 (P7) mice with ethanol (subcutaneous) and harvested the brain 6 or 24 hours post-treatment. After ethanol-induced apoptosis, we found cleaved caspase-3 is readily visible in apoptotic cell bodies (for example corpses in the cortex, not shown), as has been previously described [14]; however, only minimal signal was detected in structures known to contain degenerating axons. Rare, isolated degenerating axon fibers are detected, but overall, CC3 is not readily visible in degenerating axon tracts via standard detection techniques. Tyramide signal amplification (TSA) enables the detection of activated caspase-3 in degenerating axons. CC3 signal amplified with TSA localizes to axon tracts labeled with neurofilament in a pattern that mimics fractin and TubulinΔCsp6 (Figure 3f).

Unlike cleaved caspase-3, fractin and TubulinΔCsp6 readily highlight degenerating axons as well as corpses using standard detection methods. This suggests that while caspase 3 activity is present in degenerating axons, the concentration of cleaved caspases may be below the threshold of detection via standard detection techniques. Staining for caspase-substrate neoepitopes via fractin (Figure 3b) and TubulinΔCsp6 (Figure 3d) appears to be a more sensitive indicator of caspase activity in axons, presumably due to the much greater abundance of cleaved substrates than active enzyme.

**Figure 4:**
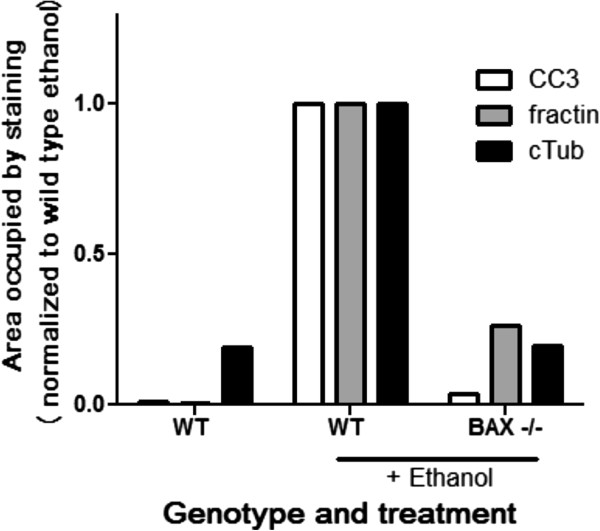
**Generation of caspase-dependent cleaved actin and tubulin neoepitopes in degenerating axons is BAX-dependent.** The amount of CC3, fractin and TubulinΔCsp6 staining was quantified 6 hours after injury via Cell profiler. Data was normalized to the value calculated from the wild type ethanol-treated tissue in order to represent relative levels in each genotype and treatment. BAX deficient mice showed nearly complete loss of ethanol-induced generation of fractin and cleaved tubulin immunoreactivity.

BAX-deficient animals are known to be protected from caspase activation in this model [14]; therefore we quantified cleaved-caspase-3, fractin and TubulinΔCsp6 staining in these mice after ethanol-induced apoptosis. We found that along with cleaved-caspase-3 staining, fractin and TubulinΔCsp6 staining is abrogated in these animals (Figure 4). This confirms that axon degeneration and production of these epitopes is downstream of a BAX-mediated, apoptosis-related process [11].

### Physiological degeneration during developmental axon pruning and during turnover of olfactory sensory neurons involves caspase-cleavage of actin and tubulin

**Figure 5:**
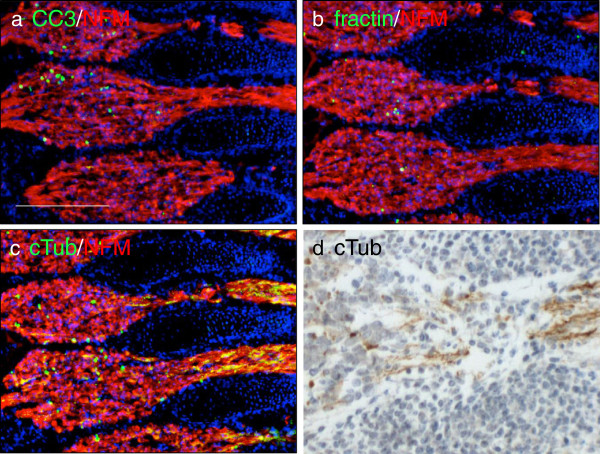
**TubulinΔCsp6 highlights degenerating axon tracts during normal developmental pruning.** Adjacent sections from embryonic day 14.5 mice were immunolabeled with CC3 **(a)**, fractin **(b)** and tubulinΔCsp6 (cTub) **(c)** (green) along with neurofilament (2H3; red). All three antibodies reveal a subset of apoptotic cell bodies in the ganglion, but only cTub prominently labels axons emanating from the ganglia **(c,d)**. Notice that cTub does not label any structures in adjacent non-neural somites (above and below axon tracts). Scale = 200 μm.

Axon degeneration occurs during developmental pruning, and we hypothesized that this degeneration also involved caspase-mediated cleavage of actin and tubulin. We harvested embryonic day 14.5 (E14.5) mice, as this is a time when we see robust apoptosis in the dorsal root ganglia, and stained for cleaved caspase-3, fractin and TubulinΔCsp6. We found prominent labeling of apoptotic corpses in the dorsal root ganglion with all the markers. However, only TubulinΔCsp6 also highlighted putative degenerating axons emanating from the ganglia (Figure 5). Relative specificity of TubulinΔCsp6 for neuronal degeneration is indicated by the fact that no labeling is seen in adjacent non-neural somites (Figure 5d).

**Figure 6:**
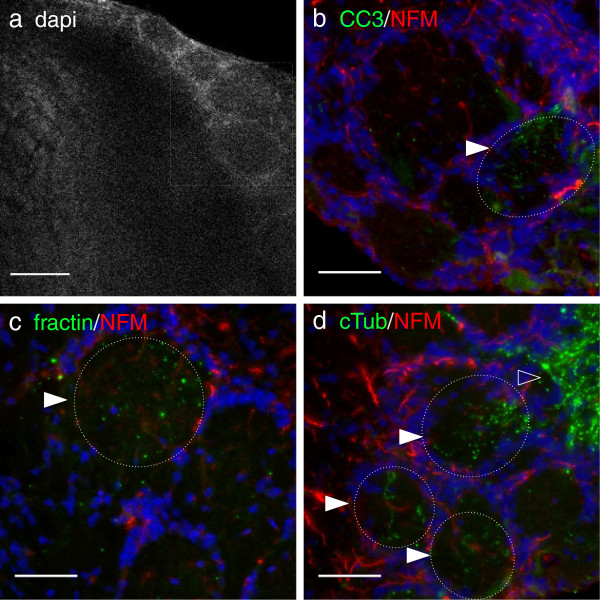
**Fractin and TubulinΔCsp6 highlight degenerating axon tracts during turnover of olfactory sensory neurons in the adult mouse brain.** Axons terminating in the glomerular layer of the adult olfactory bulb were assessed; the region with axon terminals is indicated by the box in **(a)**. Adjacent sections were stained with CC3 **(b)**, fractin **(c)** and TubulinΔCsp6 (cTub) **(d)** (green) along with neurofilament-medium (NFM; red) and the glomeruli were imaged. Fractin and TubulinΔCsp6 highlight structures consistent with fragmented neurites in a subset of glomeruli (indicated by filled arrowheads and hatched circles), likely representing neurites from olfactory sensory neurons undergoing physiological turnover. The olfactory nerve layer composed only of sensory axons, contains the highest density of TubulinΔCsp6 immunoreactive neurites (open arrowhead). Other regions of the olfactory bulb were devoid of neoepitope marker labeling. Scale bar = 50 μm.

Olfactory sensory neurons are one of the few populations known to undergo continuous turnover throughout life. The receptors and the cell bodies of olfactory sensory neurons lie in the nasal epithelium, but their axons project distally into the brain, to the olfactory bulb. The axons terminate in defined globular regions in the olfactory bulb known as glomeruli (region indicated by box in Figure 6a). Upon apoptosis of olfactory sensory neurons, their axons in the olfactory bulb glomeruli also degenerate. To our knowledge, this process has never been directly visualized *in situ*. Both fractin and TubulinΔCsp6 immunolabeling selectively highlight degenerating neurites in glomeruli and adjacent olfactory nerve layer in the adult olfactory bulb (Figure 6). Other areas of the adult mouse central nervous system that lack any known significant neuronal turnover are completely negative for these markers (data not shown).

This suggests that caspase-mediated cleavage of actin and tubulin occurs during physiological degeneration of axons in developing *and* mature nervous systems. These observations provide novel insights into the potential mechanisms of axon pruning and provide specific molecular probes to visualize these elusive events.

### Caspase-induced neoepitope markers highlight degenerating axons in human neuropathology

**Figure 7:**
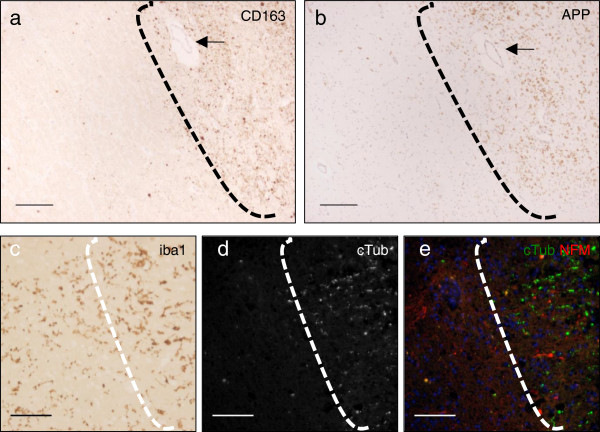
**Areas of ischemic injury in human brain are identified by infiltration of monocytes and microgliosis and there is regional overlap with markers of axon degeneration.** Adjacent sections were stained (arrow in **(a,b)** identifies the same vessel). Areas of injury (to the right of the dashed line) are identified by infiltration of monocytes **(a)**, stained by the monocyte marker CD163, as well as by a marker for degenerating axons, APP **(b)**. In addition, a microglial marker (Iba1) highlights enlarged ameboid microglia in areas of injury, which is suggestive of microglial activation **(c)**. Injured regions identified by monocyte infiltration and microgliosis also showed evidence of axon degeneration; shown here is TubulinΔCsp6 (cTub) staining **(d-e)**. Scale in **(a-b)** is 200 μm, scale in **(c-e)** is 25 μm.

**Figure 8:**
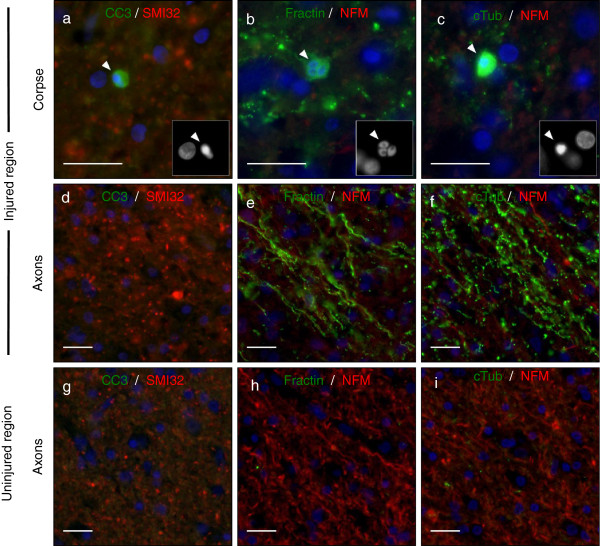
**Fractin and TubulinΔCsp6 highlight apoptotic bodies and degenerating neurites in areas of ischemic injury in human brain.** Adjacent sections were co-labeled with CC3 (green) and SMI32 (red) **(a,d,g)**, or fractin (green) and neurofilament (red) **(b,e,h)**, or TubulinΔCsp6 (cTub) and neurofilament (red) **(c,f,i)**. Areas of injury contained apoptotic neurons and degenerating axons (Row 1 and Row 2). (Row 1) Insets show DAPI-labeled pyknotic nuclei (arrowhead) in apoptotic corpses that are labeled with CC3 **(a)**, fractin **(b)** and TubulinΔCsp6 **(c)**. (Row 2) Fractin **(e)** and TubulinΔCsp6 **(f)** antibodies highlight degenerating axons, whereas CC3 **(d)** fails to label degenerating axons via conventional staining techniques. (Row 3) Uninjured areas **(g-i)** have decreased SMI32 staining and no fractin or TubulinΔCsp6-labeled axons. Scale = 25 μm.

To determine whether these antibodies may be useful as markers of neuronal apoptosis and axon degeneration in human neuropathological specimens, we obtained autopsy brain tissue from five patients who had been diagnosed with acute or subacute infarcts of the CNS (Table 1). We show representative images from an adult case (Figure 7) and an infant case (Figure 8). The most widely utilized marker of acute/subacute axonal injury, amyloid precursor protein (APP), confirmed the presence of injured axons in all five cases. We considered areas to be injured if the exhibited amoeboid IBA1-positive cells and axonal APP immunoreactivity.

**Table 1:** Cases used in analysis of axon degeneration after infarct injury

***Age***	***Diagnosis***	***Approximate stage of injury***
Prenatal (33 weeks estimated gestational age)	Placental infarction	Subacute
Newborn	Acute hypoxic injury	Acute
6 days	Cardiomyopathy, multifocal acute and subacute ischemic injury	Subacute
6 months	Small and large intestine necrosis; sepsis, watershed infarcts	Acute, 5–7 days
35 years	Sepsis, cerebral infarction	Acute, 2–5 days

We could not definitively distinguish whether the degenerating axons were confined to the area of infarct or if they were also in distal injured axon tracts. That the degenerating axons were in areas of injury was supported by CD163 immunostaining, revealing increased numbers and staining intensities of macrophage/microglia (Figure 7a). Also, these cells show activated ameboid morphology, identified via ionized calcium-binding adapter molecule 1 (Iba1) staining, (Figure 7c). This histology distinguished injured versus uninjured regions for analysis of axon degeneration.

Injured areas showed evidence of axon degeneration via our caspase cleavage-specific antibodies (Figure 7d-e, to the right of the dashed line). We found robust evidence of fractin and TubulinΔCsp6 staining in all five cases, confined to areas of axonal injury as defined by monocyte infiltration, microgliosis and APP labeling.

We found that fractin and tubulinΔCsp6 highlight apoptotic corpses (Figure 8b-c) as well as degenerating axons (Figure 8e-f). Uninjured areas were negative for these markers (Figure 8g-i). Dephosphorylation of neurofilaments is an established marker of axonal injury [25]; therefore, we compared fractin and TubulinΔCsp6 staining to CC3 and to an antibody recognizing de-phosphorylated neurofilament (antibody SMI32). We found that fractin and TubulinΔCsp6 provided a higher signal-to-noise ratio for staining degenerating axons as compared to either CC3 or SMI32 (Figure 8).

The utility of these markers is not limited to hypoxic-ischemic injury, as we also observed immunoreactivity in active acute multiple sclerosis lesions (data not shown).

## Discussion

The apoptotic cascade has been intensely studied and has been shown to be dependent on caspases, both as initiators and as executioners. During apoptosis, activated caspases are responsible for cleaving many proteins, including structural and repair proteins. Different executioner caspases, for example caspase-3 and caspase-6, have distinct, non-redundant roles [5]. Axon degeneration involves cytoskeletal degradation and morphological changes such as prominent blebbing and fragmentation. These are also hallmark features of apoptosis and, in that context, modulated by caspase activity [26]. Due to the parallels between apoptosis and axon degeneration, we hypothesized that cytoskeletal degradation in axon degeneration would similarly involve caspase activity.

Initial studies were unable to detect caspase activation in degenerating axons and suggested they had no role [9]. However, this is likely due to a lack of sensitive markers and a low threshold of caspase activation required. Indeed, our experience with cleaved-caspase-3 staining suggests activity may be below the threshold for detection via standard staining techniques. However, we find that tyramide signal amplification enables more sensitive detection of activated caspase-3 and allows for detection in degenerating axons.

Other studies argued against a role for caspases in axon degeneration because they found that caspase inhibitors were unable to prevent degeneration [9,27,28]. However, under normal circumstances inhibitors may not be potent enough, especially if there is a low threshold of caspase activation required to initiate the downstream cascade. A recent study showed that inhibitors were able to delay NGF-induced axon degeneration, however, only in caspase-3 heterozygous neurons, not wild type neurons [10].

We aimed to look further at the role of caspases in axon degeneration, and we focused on the role of caspases in cytoskeletal degradation. In order to examine whether caspase-mediated cleavage of cytoskeletal elements was involved, we used antibodies that are specific to the caspase-cleaved forms of actin and tubulin. We show via *in vitro* assays that caspases are sufficient to cleave actin and tubulin, and our cleavage-specific antibodies recognize a neoepitope generated by cleavage.

In order to determine whether apoptosis-induced axon degeneration involves caspase-mediated cytoskeletal degradation, we used an animal model of fetal ethanol syndrome. Ethanol exposure during development results in robust neuronal apoptosis [13]. Ethanol-induced neuronal death has been shown to be dependent on the intrinsic apoptotic pathway, as animals deficient in the pro-apoptotic mitochondrial proteins BAX are resistant to ethanol-induced caspase-3 activation and apoptosis [14,17]. We found that ethanol-induced apoptosis led to extensive degeneration that was detectable via the fractin and TubulinΔCsp6 antibodies. As expected, animals deficient in BAX (which are protected from intrinsic pathway caspase activation) did not generated caspase-dependent neoepitopes for fractin and TubulinΔCsp6 after ethanol treatment.

In addition, we show that antibodies against caspase-cleaved substrates not only provide specific markers that indicate caspase activity but also allow enhanced visualization of degenerating axons compared to cleaved caspase-3. Antibodies specific to caspase-cleaved actin and tubulin prove to be very useful for detecting caspase activation in axon degeneration. This is likely because a) one activated caspase molecule is able to cleave multiple substrate molecules, leading to amplified signal, and b) cytoskeletal elements are an abundant substrate in axons.

This further supports the idea that apoptosis-related mechanisms are involved in cytoskeletal degradation during axonal degeneration. Our results support a recent study that showed caspases are involved in certain types of axon degeneration [10]. They used caspase-3 and caspase-6-deficient neurons and showed that they are protected from NGF-deprivation induced axon degeneration. Our findings similarly suggest that caspases are involved in apoptosis-induced degeneration. We find caspase-cleaved tubulin and fractin in axons degenerating due to NGF-deprivation or ethanol-induced apoptosis. Overall these findings solidify a role for caspase-3 and caspase-6 as mediators of cytoskeletal degradation during axon degeneration.

We show that caspases are responsible for generating the specific cleavage products. Caspase-3 generates fractin whereas caspase-3 and caspase-6 can both generate cleaved tubulin. This specificity is useful for evaluating which caspases are involved in axon degeneration in different contexts, such as during apoptosis versus during pruning.

Our staining in the embryo sheds light on processes that occur during developmental pruning. There are some previous studies that suggest a role for caspases in pruning. A study in *Drosophila* showed that caspases are involved in developmental dendrite pruning [29]. Another study indicated that signaling of APP (amyloid precursor protein) through death receptor 6 leads to caspase-6 dependent degeneration during pruning in the mouse embryo [11]. A more recent study showed that caspase-3 and caspase-6 are necessary for proper retinocollicular pruning in mice [10]. Not surprisingly, many neurons are undergoing apoptosis at E14.5, particularly in the ganglia. As expected, cleaved-caspase-3, fractin, and TubulinΔCsp6 staining highlight the apoptotic bodies. However, the emanating fibers are preferentially highlighted by TubulinΔCsp6. This is in contrast to ethanol apoptosis-induced degeneration, where there are comparable levels of fractin and cleaved tubulin. The predominance of cleaved tubulin (produced by both caspase-3 caspase-6) and lack of fractin (produced by caspase-3) may suggest that caspase-6 is the major mediator of cytoskeletal degeneration during pruning. This validates other studies that suggest that caspase-3 and caspase-6 may have unique roles in cell body versus axon degeneration [8,11]. In addition to illuminating details about caspase activity during pruning, our finding also sheds light on the role of microtubule versus actin degradation and adds information about the role cytoskeletal degeneration during pruning.

Pruning can occur via a combination of retraction and degeneration, both of which have been well visualized in the peripheral nervous system at the neuromuscular junction [30]. However, the relative importance of each in other contexts is a bit unclear as it has been difficult to study via static techniques. While retraction may be important for modest local remodeling, removal of larger fragments appears to involve degeneration [30,31]. Retraction is speculated to depend largely on actin dynamics, whereas degeneration involves microtubules [3]. The predominance of cleaved tubulin in the fibers supports the idea that a) caspase-6-mediated tubulin cleavage occurs pruning and b) that degeneration is occurring as opposed to just retraction.

We were interested in determining whether these antibodies may be useful as markers of neuronal apoptosis and axon degeneration in human pathological specimens. We found that fractin and TubulinΔCsp6 antibodies highlight acute axonal degeneration in neonatal, infant, and adult hypoxic-ischemic injury as well as in a sample of an active multiple sclerosis lesion. Activated caspase-6 has been detected in corpses after ischemic injury [32]. Another paper showed fractin in hypoxic-ischemic injury, and they do mention finding positive axon segments [33]. We also stained tissue from patients with a variety of chronic neurodegenerative diseases, including Alzheimer’s disease, Parkinson’s disease and epilepsy, and did not find that these markers specifically highlighted a significant amount of axonal debris in these tissues. Others have shown fractin and TubulinΔCsp6 staining in tissue from Alzheimer’s disease in Hirano bodies and neuronal bodies respectively [22,34], but they do not note labeling of axon degeneration. The mechanisms of axon degeneration in these other diseases have not been well elucidated, and it is difficult to conclude precisely why we found that these cases are negative for these markers. It is possible that the degeneration is not caspase-dependent, but it is also possible that there are not enough simultaneously degenerating neurons to detect significant signal at an isolated time point. Ultimately, our work to date suggests that fractin and TubulinΔCsp6 antibodies are best used as markers of acute/subacute degeneration.

While fractin and cleaved tubulin staining can serve as readouts of caspase-3 versus caspase-6 activity, one must keep in mind the possible limitations of these markers. First of all, staining is dependent on whether the proteins localize to and are abundant in the compartment of interest as well as whether the specific caspases are activated. Axons are rich in actin and tubulin, therefore these are good caspase substrates to use to study caspase-mediated cytoskeletal degradation in axons. Secondly, as degeneration progresses, these epitopes may be degraded and no longer recognized by these markers, i.e. the lifespan of the epitope may be limited. Despite these limitations, we have found these markers to be very useful.

There is potential for application of these markers in forensic pathology. The current standard is APP [35]. APP is limited in its ability to specifically identify injury-induced degeneration [36]. Also, there is interest in identifying specific axon degeneration markers to indicate details such as etiology of pathology. APP has limitations, for example in differentiating between trauma and ischemic injury [37,38]. It would be of interest to determine whether fractin and TubulinΔCsp6 patterns differ in ischemia compared to trauma.

Several important mechanistic questions remain unanswered. Is caspase-mediated degradation of the cytoskeleton directly involved in mediating axon blebbing and fragmentation? There is support for this hypothesis, as one study showed that exogenous expression of actin fragments (caspase-cleaved size fragments) was sufficient to induce morphological changes of apoptosis downstream of caspase activation [39]. This could also be examined by testing whether mutation of the caspase-cleavage sites on actin and tubulin confers any resistance to blebbing and fragmentation of degenerating axons. What alternative pathways can mediate cytoskeletal degradation and axonal degeneration when caspases may not be the sole executioners, as in Wallerian degeneration? Calpains are likely candidates, as they have been shown to be activated after axotomy and inhibiting calpains confers resistance to axotomy-induced degeneration [2,40].

## Conclusions

In conclusion, caspase-mediated cleavage of actin and tubulin is a common feature of several forms of axonal degeneration. Antibodies specifically recognizing cleaved proteins serve as useful markers of caspase activity and design of additional antibodies targeting specific caspase-generated neoepitopes would provide valuable tools for the field. In particular, it would be useful to identify caspase-substrate neoepitopes specific for glial and neuronal subtypes and for subcellular compartments, including dendrites, axons or synapses.

## Competing interests

The authors declare that they have no competing interests.

## Authors’ contributions

JDS carried out the mouse ethanol apoptosis experiments, performed immunolabeling, did quantitative image analysis and wrote large portions of the manuscript. KKG performed in vitro NGF-deprivation experiments. DSF performed tissue processing, immunostaining, western blotting and prepared portions of figures for the manuscript. ACL provided recombinant activated caspases and advice and protocols on the in vitro experiments, and provided antibodies to caspase-cleaved tubulin. CDD oversaw the NGF-deprivation experiments and provided microfluidic chambers and advice on use of this model to study axon degeneration. JWM oversaw all of the experiments, prepared some of the figures, and wrote and edited portions of the manuscript. All authors read and approved the final manuscript.
